# Circular RNA circ_0062389 modulates papillary thyroid carcinoma progression via the miR-1179/high mobility group box 1 axis

**DOI:** 10.1080/21655979.2021.1914470

**Published:** 2021-04-29

**Authors:** Yujuan Wang, Huafeng Zong, Haicheng Zhou

**Affiliations:** a Otorhinolaryngology Head and Neck Surgery, Shaanxi Provincial People’s Hospital, Xi’an, Shaanxi, China; b Department of Pathology, Dalian Friendship Hospital, Dalian, Liaoning, China; c Department of Endocrinology, The First Affiliated Hospital of Dalian Medical University, Dalian, Liaoning, China

**Keywords:** Papillary thyroid carcinoma, circ_0062389, miR-1179, HMGB1

## Abstract

Circular RNAs (circRNAs) feature prominently in regulating the progression of tumors, including papillary thyroid carcinoma (PTC). This work is designated to delve into the role of circ_0062389 in PTC. Generally, quantitative real-time polymerase chain reaction (qRT-PCR) was employed to detect circ_0062389, miR-1179 and high mobility group box 1 (HMGB1) mRNA expression levels. RNase R assay was used to verify the circular characteristics of circ_0062389. After circ_0062389 was knocked down in PTC cells, CCK-8 assay was adopted to determine cell viability. Wound healing assay was leveraged to probe cell migration. Besides, Western blot assay was executed to examine the expression levels of HMGB1 and epithelial-mesenchymal transformation (EMT)-related markers (E-cadherin and N-cadherin). Dual-luciferase reporter assay was performed to authenticate the targeting relationships between miR-1179 and circ_0062389, as well as miR-1179 and HMGB1. Here, this work proved that circ_0062389 was greatly up-regulated in PTC tissues and cell lines. The high expression of circ_0062389 was related to large tumor size and positive lymphatic metastasis. Knocking down circ_0062389 could inhibit the proliferation, migration and EMT process of PTC cells. Besides, miR-1179 was a downstream molecule of circ_0062389. Furthermore, miR-1179 inhibitors could partially reverse the above effect of knocking down circ_0062389 on PTC cells. It was also confirmed that HMGB1 was a direct target of miR-1179 and mediated the effects of circ_0062389 and miR-1179 in PTC. Altogether, circ_0062389 can adsorb miR-1179, and regulate HMGB1 expression, thus playing a role in PTC.

## Graphical abstract

**Figure:**
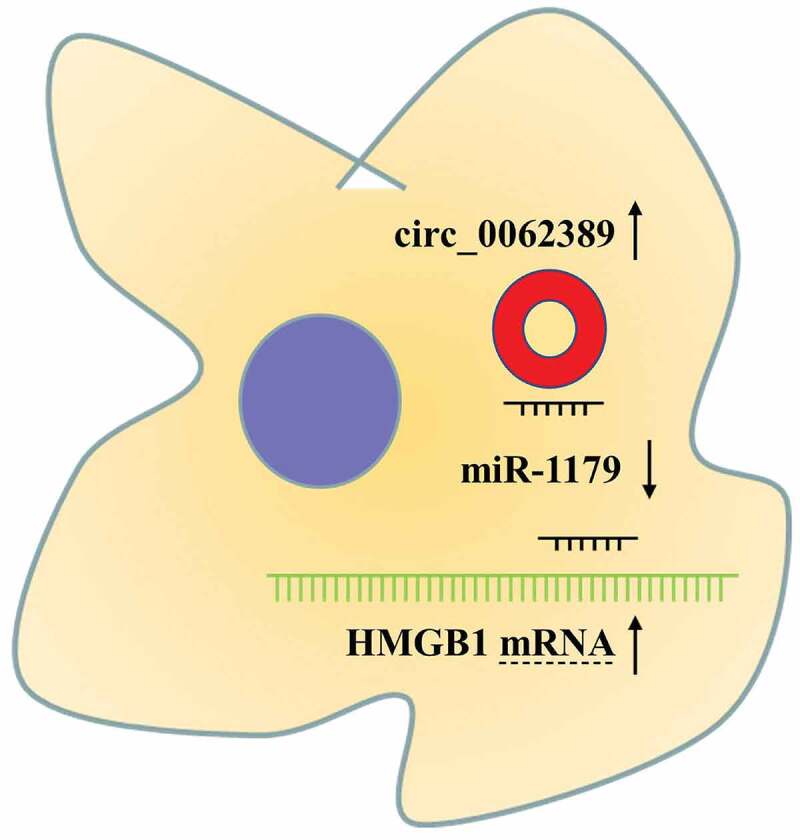

## Introduction

Papillary thyroid cancer (PTC) is a common tumor, accounting for about 80%-85% of thyroid cancer cases [1]. The 10-year survival rate of PTC patients exceeds 90% [2]. However, some PTC patients still have a poor prognosis due to their older age, larger primary tumor, distant metastasis and lymph node metastasis [3]. It is necessary to further improve the treatments of PTC, and it is of great significance to explore the pathogenesis of PTC for finding new targets for PTC treatment.

Circular RNAs (circRNAs) are non-coding RNAs with a covalently closed circular structure, without 5ʹ cap or 3ʹ poly A tail [4]. CircRNA abnormal expression has been confirmed to be involved in many human diseases, especially tumors [5]. Reportedly, many circRNAs are implicated in PTC progression, such as circRNA_102002, circ_LDLR and circPRMT5^6-^[6]. Circ_0062389, as a member of circRNAs, regulates CCNE1 expression by adsorbing miR-103a-3p to promote the progression of non-small cell lung cancer (NSCLC) [7]. However, the function and molecular mechanism of circ_0062389 in the progression of PTC are still indeterminate.

MicroRNAs (miRNAs), non-coding RNAs with about 22 nucleotides, regulate gene expression via interacting with the 3ʹ untranslated region (3ʹUTR) of mRNA [8]. Many miRNAs, as reported, are involved in PTC progression, such as miR-224-5p, miR-188-5p and miR-451a [9–11]. In addition, miR-1179 can affect the progression of various tumors, such as oral cancer, gastric cancer and breast cancer [12–14]. In PTC, miR-1179 can target HMGB1 or ABCA9 to inhibit PTC progressions [15,16]. However, the mechanism of miR-1179 dysregulation in PTC has not been completely elaborated.

High Mobility Group Box 1 (HMGB1) is involved in cancer cell proliferation, autophagy, metastasis and epithelial-mesenchymal transition (EMT) via regulating various signaling pathways [17]. Reportedly, HMGB1 participates in the progression of many diseases, including PTC [17]. However, the mechanism of HMGB1 dysregulation in PTC still awaits more verification.

This work aims to explore the expression characteristics and underlying mechanism of circ_0062389 in PTC. It was hypothesized that circ_0062389 was up-regulated in PTC tissues, and accelerate PTC cell growth, migration and EMT via modulating miR-1179/HMGB1 axis, and the experiments were performed to validate the scientific hypothesis mentioned above.

## Materials and methods

### Participants and tissue samples

Paired PTC tissues and adjacent tissues (n = 58) were available from Dalian Friendship Hospital, and all samples, stored at −80°C immediately after removal to avoid RNA loss, were confirmed by pathologists. None of the patients had received radiotherapy, chemotherapy or other treatment before the surgery. This study, with signed written informed consent from the patients, was approved by the Ethics Committee of Dalian Friendship Hospital.

### Cell culture and transfection

Human PTC cell lines (K-1, SW1736, TPC-1, SW579) and human thyroid follicular epithelial cells Nthy-ori 3–1 were available from Cell Bank of Chinese Academy of Sciences (Shanghai, China). Notably, all cells were accordingly cultured in Roswell Park Memorial Institute (RPMI)-1640 medium with 10% fetal bovine serum (FBS), 100 IU/mL penicillin and 100 μg/mL streptomycin. The above materials or reagents were from Gibco (Carlsbad, CA, USA).

Small interfering RNA (siRNA) oligonucleotides targeting circ_0062389 (si-circ_0062389#1: 5ʹ-TGCAGACCTCCCGACCTCTTT-3ʹ, si-circ_0062389#2: 5ʹ-ATCTGCTGTCTGCAGACCTCC-3ʹ and si-circ_0062389#3: 5ʹ-GATCTGCTGTCTGCAGACCTC-3ʹ), miR-1179 mimics (5ʹ-AAGCAUUCUUUCAUUGGUUGG-3ʹ) and miR-1179 inhibitors (miR-1179 in, 5ʹ- CCAACCAAUGAAAGAAUGCUU-3ʹ) and their corresponding control (miR-NC, 5ʹ-UCACAACCUCCUAGAAAGAGUAGA-3ʹ) were synthesized by Genomeditech (Shanghai, China). The above vector plasmids or oligonucleotides were respectively transfected into PTC cell lines by lipofectamine 2000 (Invitrogen, Carlsbad, CA, USA) when the cells reached 80% confluence.

### Quantitative real-time polymerase chain reaction (qRT-PCR) and RNase R digestion assay

20 mg/ml RNase R (Genesee, Guangzhou, China) was incubated with 10 μg RNA at 37°C for 20 min, and a control group without RNase R treatment was set. Total RNA was extracted from tissues and cell lines by TRIzol reagent (Invitrogen, Carlsbad, CA, USA), and reverse transcription was performed with PrimeScript™ RT reagent Kit (Takara, Dalian, China). Following that, a SYBR Green Premix Ex TaqTM kit (Takara, Kusatsu, Japan) was adopted to perform qRT-PCR, with relative RNA expressions calculated by 2^−ΔΔCt^ formula, and glycerol aldehyde-3-phosphate dehydrogenase (GAPDH) and U6 were regarded as internal references. Primer sequences are detailed in Table 1.

**Table 1.** Primer sequences

	Forward	Reverse
circ_0062389	5’- TCTGAGAAGCTGCAGTCCAA-3’	5ʹ- TCTGAGAAGCTGCAGTCCAA-3’
miR-1179	5ʹ-GCGGAAGCATTCTTTCAT-3’	5ʹ- CAAGGGCTCGACTCCTGT-3’
PI4KA	5ʹ-AGCTCCGCAGCACTATCATC −3’	5′-TCCATACACCCCAAGAGTTGTG-3’
HMGB1	5ʹ-GTGAACTGCTGCACGAAGAA-3’	5ʹ- GCCTTTGAAATGTGCTCCCA-3’
U6	5ʹ-GACTATCATATGCTTACCGT-3’	5ʹ-GGGCAGGAAGAGGGCCTAT-3’
GAPDH	5’-CTTTGGTATCGTGGAAGGACTC-3’	5ʹ-GTAGAGGCAGGGATGATGTTCT-3’

### Cell counting kit-8 (CCK-8) assay

Cell proliferation was detected by CCK-8 assay. PTC cells were inoculated on a 96-well plate at a density of 2 × 10^3^ cells/well and cultured, and 10 µL of CCK-8 solution (Solarbio, Beijing, China) was added into each well at 0 h, 24^th^ h, 48^th^ h, and 72^th^ h. And the cells were accordingly incubated for another 4 h. Ultimately, the absorbance at 450 nm was measured and recorded by a microplate reader (Thermo-Fisher Scientific, Waltham, MA, USA).

### Wound healing assay

Transfected cells were inoculated on a 6-well plate. When the cells reached 90% confluence, a cell-free region was created by scraping the monolayer cells with a 200 μL micropipette. Cells were cultured in serum-free medium, and the wound closure was measured at 0 h and 24^th^ h. Wound width (%) = (0 h wound closure-24^th^ h wound closure)/0 h wound closure ×100%。

### Bioinformatics analysis

StarBase database (starbase.sysu.edu.cn) was used to predicted the binding sites between circRNA and miRNA, as well as miRNA and mRNA.

### Dual-luciferase reporter assay

Wild-type and mutant reporter plasmids of circ_0062389 or HMGB1 3ʹUTR containing the putative binding sequence with miR-1179 were designed and constructed by Promega (Madison, WI, USA). Above luciferase reporters were respectively co-transfected with miR-1179 mimics or miR-NC into PTC cells, and the dual-luciferase reporter assay system (Promega, Madison, Wis., USA) was utilized to detect the luciferase activity of the cells in each group after 48 h.

### Western blot assay

Total protein was respectively extracted from cells in different groups by radio-immunoassay assay (RIPA) lysis buffer (Beyotime, Shanghai, China), with the concentration of protein quantified by a bicinchoninic acid (BCA) detection kit (Beyotime, Shanghai, China). 30 μg protein samples in each group were subjected to sodium dodecyl sulfate-polyacrylamide gel electrophoresis (SDS-PAGE) and then transferred to polyvinyl fluoride (PVDF) membranes (Millipore, Billerica, MA, USA), which was then blocked with 5% skimmed milk. Then the PVDF membrane was incubated with primary antibodies anti‐HMGB1 (1:1000, ab18256, Abcam Inc., Cambridge, UK), anti‐E-cadherin (1:1000, ab40772, Abcam Inc., Cambridge, UK), anti‐N-cadherin (1:1000, ab98952, Abcam Inc., Cambridge, UK) or anti-GAPDH (1:1000, ab9485, Abcam Inc., Cambridge, UK) overnight at 4°C, and then incubated with the horseradish peroxidase-conjugated secondary antibody (1:2000, ab150077, Abcam Inc., Cambridge, UK) at ambient temperature for 1 h, and finally the ECL luminescence reagent (ThermoFisher Scientific, Waltham, MA, USA) was employed to detect the protein bands.

### Statistical analysis

All experiments were repeated for three times before collecting data, with results expressed as ‘mean ± standard deviation’. SPSS17.0 software (SPSS Inc., Chicago, IL, USA) was used for statistical analysis and GraphPad Prism V5.0 (GraphPad Software, Inc., La Jolla, CA, USA) was used for drawing the plots. The PTC patients were grouped into circ_0062389 high expression group and circ_0062389 low group with the median expression of circ_0062389 as the threshold. The correlation between circ_0062389 expression and the clinicopathological characteristics of the patients was analyzed by Chi-square test. Student’s *t* test was adopted for difference analysis between two groups, and one-way analysis of variance was adopted for difference analysis among three or more groups. Pearson correlation was adopted for correlation analysis. Statistically, *P < 0.05, **P < 0.01, and ***P < 0.001 were meaningful.

## Results

The aim of this work was to explore the expression pattern and function of circ_0062389 in PTC, and it was demonstrated that that circ_0062389 expression was raised in PTC tissues and cell lines. We also reported that, circ_0062389 could promote PTC cell viability, migration and invasion by sponging miR-1179 and upregulating the expression of HMGB1.

### The expression and characteristics of circ_0062389 in PTC

**Figure 1.**
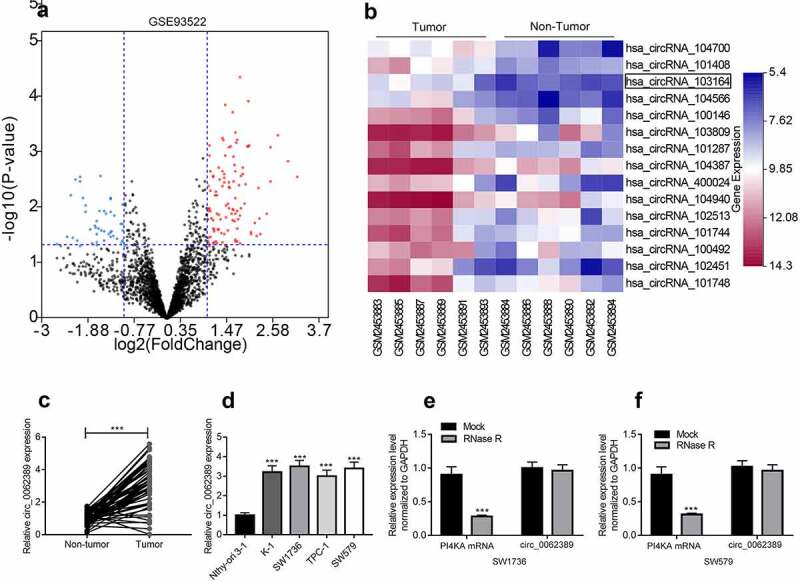
***Circ_0062389 is significantly up-regulated in PTC tissues and cell lines*** (a)&(b) Volcanic and heat maps showed that multiple circRNAs, including Circ_0062389, were differentially expressed in PTC tissues and adjacent tissues in GSE93522; (c) qRT-PCR was used to detect the expression of circ_0062389 in PTC tissues and adjacent tissues; (d) qRT-PCR was performed to evaluate the expression of circ_0062389 in PTC cell lines and normal cell line; (e)&(f) After RNase R treatment, qRT-PCR was used to detect the expression of circ_0062389 and PI4KA mRNA in total RNA. ***P < 0.001

To identify PTC-related circRNAs, a circRNA microarray (GSE93522) was analyzed to identify the differentially expressed circRNAs in 6 pairs of PTC tissues and adjacent tissues. According to the criteria (fold-change > 2.0 and *P* value < 0.05), heat maps and volcano plots were drawn, and the results showed that circ_103164 (also known as circ_0062389) expression was dramatically raised in PTC tissues (Figure 1a&B). Then, qRT-PCR also showed that in 58 PTC patients, circ_0062389 expression in PTC tissues was markedly higher than that in adjacent tissues (Figure 1c) and the high expression of circ_0062389 was related to large tumor size and positive lymph node metastasis (Table 2). Circ_0062389 expression level in thyroid cancer cell lines (K-1, SW1736, TPC-1, SW579) was also significantly higher compared with that in Nthy-ori 3–1 cell line (Figure 1d). Considering the highest expression of circ_0062389 in SW1736 and SW579, these two cell lines were selected for the follow-up functional experiments.

**Table 2.** The relationship between circ_0062389 expression and clinicopathological features

Features	n	circ_0062389		*P*
		High (n = 29)	Low (n = 29)	
Age (years)				
≥60	28	13	15	0.599
<60	30	16	14	
Gender				
Male	35	18	17	0.788
Female	23	11	12	
Lymphatic metastasis				
Positive	30	19	11	0.035
Negative	28	10	18	
Tumor size (cm)				
≥2	34	21	13	0.032
<2	24	8	16

In addition, RNase R was used to treat total RNA and verify the circular character of circ_0062389. The results showed that circ_0062389 was resistant to RNase R digestion, whereas the linear PI4KA mRNA was degraded by RNase R (Figure 1e&F). Therefore, it was revealed that circ_0062389 was stable in PTC and may be related to the progression of PTC.

### Circ_0062389 can promote the multiplication, migration and EMT of PTC cells

**Figure 2.**
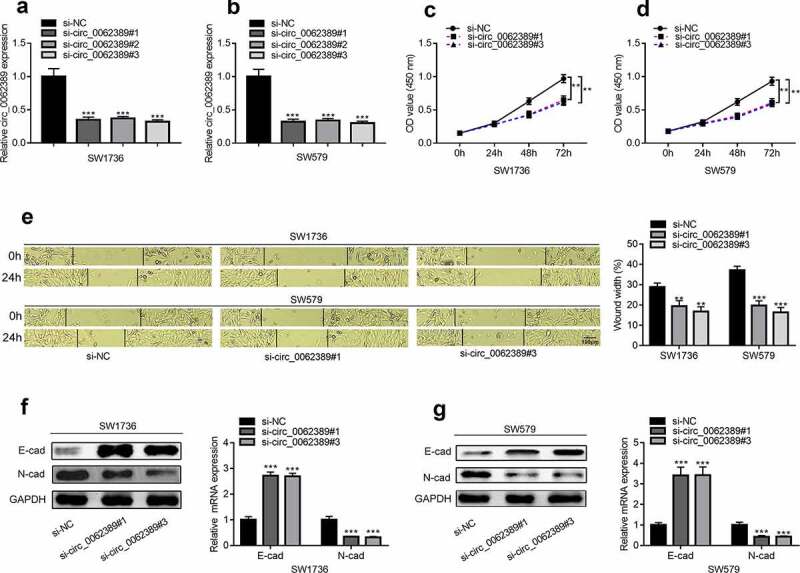
***Circ_0062389 can promote the proliferation, migration and EMT process of PTC cells*** (a)&(b) qRT-PCR was used to detect the expression of circ_0062389 after transfection of three circ_0062389 siRNA into PTC cells; (c)&(d) CCK-8 assay was used to evaluate the proliferation of PTC cells transfected with circ_0062389 siRNA; (e) Wound healing assay was adopted to measure cell migration in PTC cells after transfection of circ_0062389 siRNA; (f)&(g) qRT-PCR and Western blot assay were used to detect the expression of E-cadherin and N-cadherin at mRNA and protein levels in PTC cells transfected with circ_0062389 siRNA. **P < 0.01, and ***P < 0.001

Considering that circ_0062389 was elevated in PTC tissues and cell lines, loss-of-function experiments were adopted to explore the function of circ_0062389 in PTC. Three circ_0062389 siRNAs (si-circ_0062389#1, si-circ_0062389#2 and si-circ_0062389#3) were transfected into SW1736 and SW579 cell lines, and the results showed that circ_0062389 siRNAs significantly decreased circ_0062389 expression in SW1736 and SW579 cell lines, and si-circ_0062389#1 and si-circ_0062389#3 had higher transfection efficiency, which were used for subsequent experiments (Figure 2a&B). CCK-8 assay uncovered that knocking down circ_0062389 in SW1736 and SW579 cell lines could significantly inhibit cell proliferation (Figure 2c&D). As against si-NC group, the ability of cell migration in si-circ_0062389#1 and si-circ_0062389#3 groups was significantly reduced (Figure 2e). Also, qRT-PCR and Western blot assays proved that circ_0062389 depletion significantly increased E-cadherin protein expression and decreased N-cadherin protein expression (figure 2f&G). These data confirmed that knocking down circ_0062389 could inhibit the growth, migration and EMT of PTC cells.

### Circ_0062389 is the molecular sponge of miR-1179 in PTC cells

**Figure 3.**
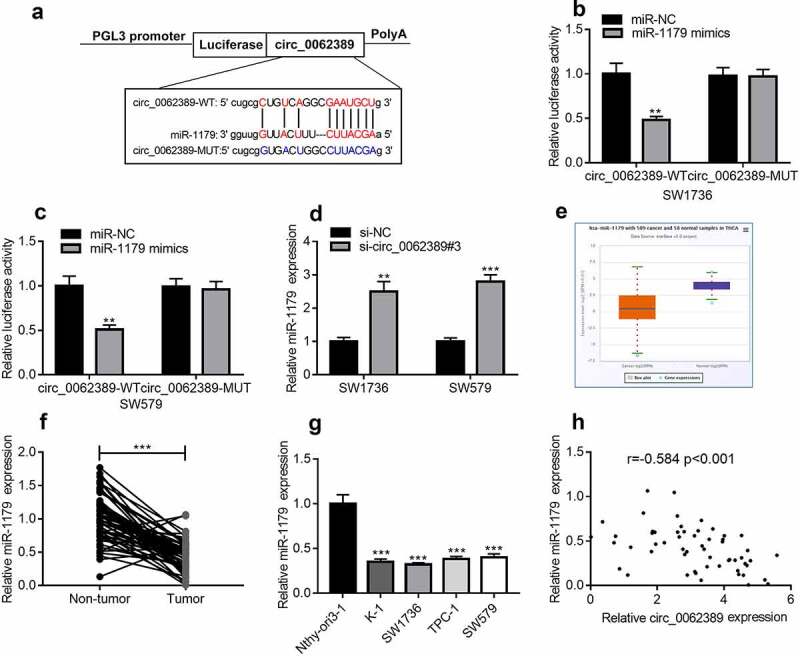
***MiR-1179 is a target of circ_0062389*** *(a)*StarBase database predicted that there was a binding site between miR-1179 and circ_0062389; (b)&(c) Dual-luciferase reporter gene assay confirmed that miR-1179 could negatively regulate the luciferase activity of circ_0062389-WT, rather than that of circ_0062389-MUT; (d) qRT-PCR was used to detect miR-1179 expression after PTC cells were transfected with circ_0062389 siRNA; (e) StarBase database was adopted to analyze miR-1179 expression in PTC tissue and normal tissue; (f) qRT-PCR was used to detect the expression of miR-1179 in PTC tissues and adjacent tissues; (g) qRT-PCR was used to detect miR-1179 expression in PTC cell lines and normal cell line; (h) The correlation analysis of circ_0062389 and miR-1179 expression in PTC tissues. **P < 0.01; and ***P < 0.001

StarBase database showed that circ_0062389 contained potential complementary binding sites with miR-1179 (Figure 3a). Dual-luciferase reporter gene assay highlighted that miR-1179 mimics could decrease the luciferase activity of wild type circ_0062389 reporter plasmid, but did not significantly change that of mutant circ_0062389 reporter plasmid (Figure 3b&C). In addition, circ_0062389#3 with the highest knock-down efficiency was transfected into SW1736 and SW579 cell lines, and it was found that circ_0062389 inhibition significantly increased miR-1179 expression (Figure 3d). StarBase database confirmed that miR-1179 was dramatically declined in PTC tumor tissues relative to normal tissues (Figure 3e). qRT-PCR also found that miR-1179 expression in PTC tissues was greatly lower than that in adjacent tissues (figure 3f), and its expression in PTC cell lines was also markedly lower than that in normal cell line (Figure 3g). Pearson correlation analysis confirmed that miR-1179 expression level was negatively correlated with circ_0062389 expression level in PTC tissues (Figure 3h). Thus, our data indicated that circ_0062389 was the molecular sponge of miR-1179 in PTC.

### Circ_0062389 participates in PTC through miR-1179

**Figure 4.**
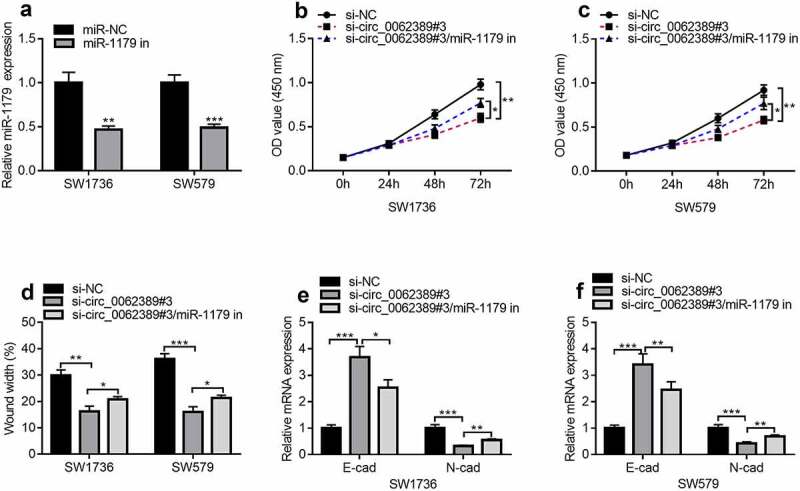
***Circ_0062389 exerts promoting effects in PTC through modulating miR-1179.*** (a) qRT-PCR was used to detect the expression of miR-1179 after transfection of miR-1179 inhibitors into PTC cells; (b)&(c) CCK-8 assay was used to detect the proliferation of PTC cells after co-transfection of circ_0062389 siRNA and miR-1179 inhibitors; (d) Wound healing assay was used to detect PTC cell migration after co-transfection of circ_0062389 siRNA and miR-1179 inhibitors into PTC cells; (e)&(f) qRT-PCR was used to detect the mRNA expression of E-cadherin and N-cadherin after co-transfection of circ_0062389 siRNA and miR-1179 inhibitors into PTC cells. *P < 0.05, **P < 0.01, and ***P < 0.001

Next, whether circ_0062389 could play a role through miR-1179 was further explored. MiR-1179 inhibitors were transfected into SW1736 and SW579 cell lines and the results uncovered that miR-1179 inhibitors could dramatically reduce the expression of miR-1179 (Figure 4a). Then circ_0062389 siRNA and miR-1179 inhibitors were co-transfected into SW1736 and SW579 cell lines. CCK-8 assay showed that miR-1179 inhibitors could partially reverse the effect of knocking down circ_0062389 on cell proliferation (Figure 4b&C). Wound healing assay uncovered that co-transfection of circ_0062389 siRNA and miR-1179 inhibitors markedly increased cell migration compared with si-circ_0062389#3 group (Figure 4d). qRT-PCR confirmed that miR-1179 inhibitors could partially reverse the increase of E-cadherin mRNA expression and the decrease of N-cadherin mRNA expression caused by knocking down circ_0062389 (Figure 4e&F). The aforementioned findings revealed that circ_0062389 could regulate PTC cell growth, migration and EMT via modulating miR-1179.

### Circ_0062389 regulates HMGB1 expression through miR-1179

**Figure 5.**
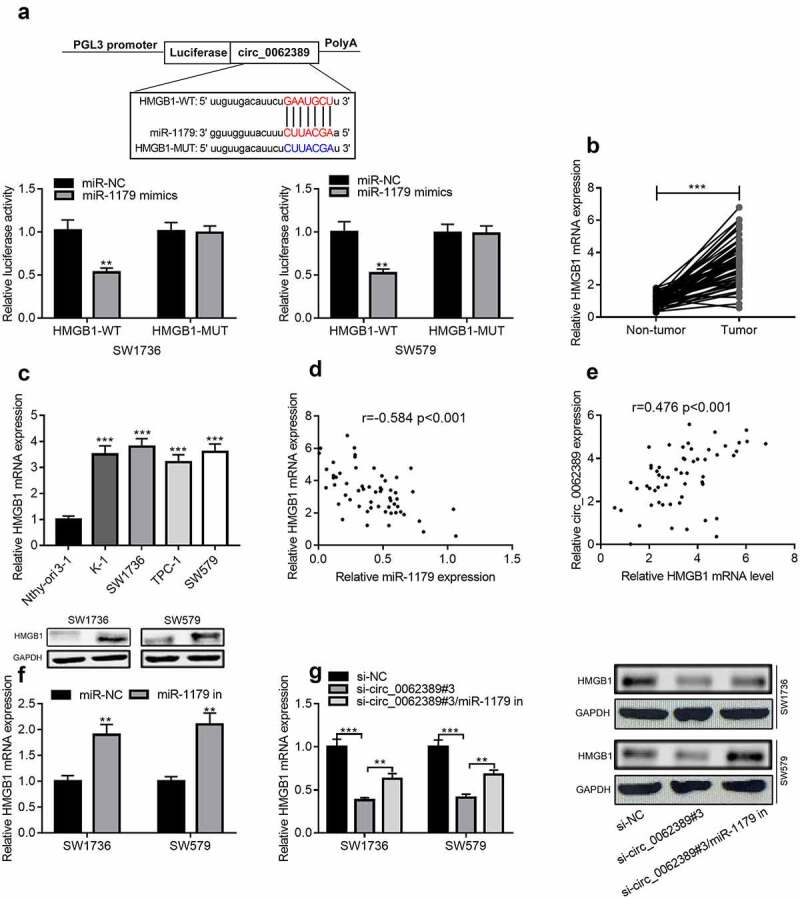
***Circ_0062389 can adsorb miR-1179 to regulate HMGB1 expression*** (a) StarBase database predicted that circ_0062389 and miR-1179 had a complementary binding site, and dual-luciferase reporter gene asasy confirmed that circ_0062389 could bind with miR-1179 directly; (b) qRT-PCR was used to evaluate the expression of HMGB1 mRNA in PTC tissues and adjacent tissues; (c) qRT-PCR was used to detect HMGB1 mRNA expression in PTC cell lines and human normal thyroid follicular epithelial cell line; (d)&(e) The correlation analysis of miR-1179 and HMGB1 mRNA, HMGB1 mRNA and circ_0062389 in PTC tumor tissues; (f)&(g) qRT-PCR and Western blot assay were used to detect the expression of HMGB1 mRNA and protein in PTC cells after transfection of miR-1179 inhibitors and co-transfection of circ_0062389 siRNA and miR-1179 inhibitors. **P < 0.01, and***P < 0.001

StarBase database was used to predict the downstream target of miR-1179, and HMGB1 3ʹUTR contained a binding site complementary to miR-1179. Dual-luciferase reporter gene assay confirmed that miR-1179 mimics could greatly reduce the luciferase activity of wild-type HMGB1 reporter plasmid, but had no obvious impact on that of mutant HMGB1 reporter plasmid (Figure 5a). qRT-PCR confirmed that HMGB1 mRNA was markedly raised in PTC tissues and cell lines (Figure 5b&C). Pearson correlation analysis highlighted that HMGB1 mRNA expression level was negatively correlated with miR-1179 expression level, and positively correlated with circ_0062389 expression level (Figure 5d&E). Additionally, miR-1179 inhibitors could significantly increase HMGB1 mRNA and protein expression levels (figure 5f). In addition, miR-1179 inhibitors could partially reverse the inhibitory effect of knocking down circ_0062389 on HMGB1 mRNA and protein expression levels (Figure 5g). Therefore, the above experiments confirmed that circ_0062389 regulated HMGB1 expression through repressing miR-1179 in PTC cells.

## Discussion

CircRNAs, as reported, are pivotal in different biological processes, especially in tumorigenesis, progression and metastasis. CircRNAs may be promising biomarkers and targets in tumor diagnosis and treatment [18]. Recently, as reported, circRNAs exert important regulatory parts in PTC [6,7,19,20]. For example, circRNA_102002 promotes PTC metastasis by regulating miR-488-3p/HAS2 axis [19]; circ_LDLR promotes PTC progression via modulating miR-195-5p/LIPH axis [20]; circPRMT5 promotes PTC cell multiplication and invasion via regulating miR-30 c/E2F3 axis [6]. Circ_0062389 expression level is significantly raised in NSCLC and can promote cell proliferation and accelerate cell cycle progression [7]. Here, the present study showed that circ_0062389 was remarkably up-regulated in PTC tumor tissues and cell lines, being resistant to RNase R digestion. The high expression of circ_0062389 was related to large tumor size and positive lymph node metastases. In addition, knocking down circ_0062389 could significantly inhibit the viability (**P < 0.01), migration (**P < 0.01) and EMT (***P < 0.001) of PTC cells. Taken together, these results confirm that circ_0062389 is structurally stable and is a cancer-promoter in the PTC progression.

CircRNAs can adsorb miRNAs and play a role as a competitive endogenous RNA (ceRNA) in PTC [21]. In the present study, StarBase database and dual-luciferase reporter gene assay proved that miR-1179 could combine with circ_0062389. MiR-1179 is down-regulated in many kinds of tumors, and blocks cancer progression. For example, in cervical cancer, circ_0084927 can adsorb miR-1179 to regulate CDK2 expression, promote cell proliferation and inhibit apoptosis [22]. MiR-1179 expression is markedly declined in oral cancer, and it can inhibit cell proliferation, enhance vincristine sensitivity and induce apoptosis by regulating MEK/ERK and PI3K/AKT signaling pathways [12]. In gastric cancer, miR-1179 restrains cell proliferation by targeting HMGB1^15^. Here, it was demonstrated that miR-1179 was remarkably decreased in PTC tumor tissues and cell lines, and was negatively correlated with circ_0062389 expression; miR-1179 inhibitors could partially reverse the suppressing effect of knocking down circ_0062389 on the malignant phenotypes of PTC cells. Therefore, it is concluded that circ_0062389 adsorbs miR-1179 in PTC, thus participating in PTC progression.

StarBase database and dual-luciferase reporter assay showed that HMGB1 was the target of miR-1179. HMGB1, a nuclear protein that exists in almost all eukaryotic cells, consists of 215 amino acid residues, and features prominently in maintaining the stability of genome structure [23–25]. Importantly, HMGB1 is a crucial regulator in cancer biology. Reportedly, HMGB1 can promote the invasion of NSCLC cells by activating PI3K/Akt and NF-κB pathways to produce MMP-9[24]; in metastatic pancreatic ductal adenocarcinoma, HMGB1 promotes EMT and invasion of cancer cells by acting on RAGE/NF-κB axis. In this work, it was observed that HMGB1 was dramatically raised in PTC tumor tissues and cell lines, and HMGB1 mRNA expression was negatively correlated with miR-1179 expression and positively correlated with circ_0062389 expression in PTC tumor tissues. In PTC cells, miR-1179 could negatively regulate HMGB1 expression, and miR-1179 inhibitors can partially reverse the inhibitory effect of knocking down circ_0062389 on HMGB1 expression. Therefore, circ_0062389 regulates HMGB1 expression via adsorbing miR-1179, to exert its regulatory function on the malignant phenotypes of PTC cells.

## Conclusion

Collectively, circ_0062389 expression level is significantly raised in PTC tissues and cell lines, and circ_0062389 participates in PTC progression as a tumor-promoter. Mechanistically, circ_0062389 can adsorb miR-1179 and up-regulate HMGB1. This study may provide a theoretical basis for further exploring biomarkers and therapy targets for PTC diagnosis and treatment.

## Supplementary Material

Supplemental Material

## Data Availability

The data used to support the findings of this study are available from the corresponding author upon request.

## Highlights

· Circ_0062389 was up-regulated in PTC tissues and cell lines.

· Circ_0062389 could promote the proliferation, migration and epithelial-mesenchymal transition (EMT) of PTC cells.

· Circ_0062389 regulated HMGB1 expression through miR-1179.
