# Supplementary figures and images for: Circular RNA circ_0062389 modulates papillary thyroid carcinoma progression via the miR-1179/high mobility group box 1 axis
Source: Bioengineered. 2021 Apr 29;12(1):1484–94. doi: 10.1080/21655979.2021.1914470 (PMC8806330; doi:10.1080/21655979.2021.1914470)

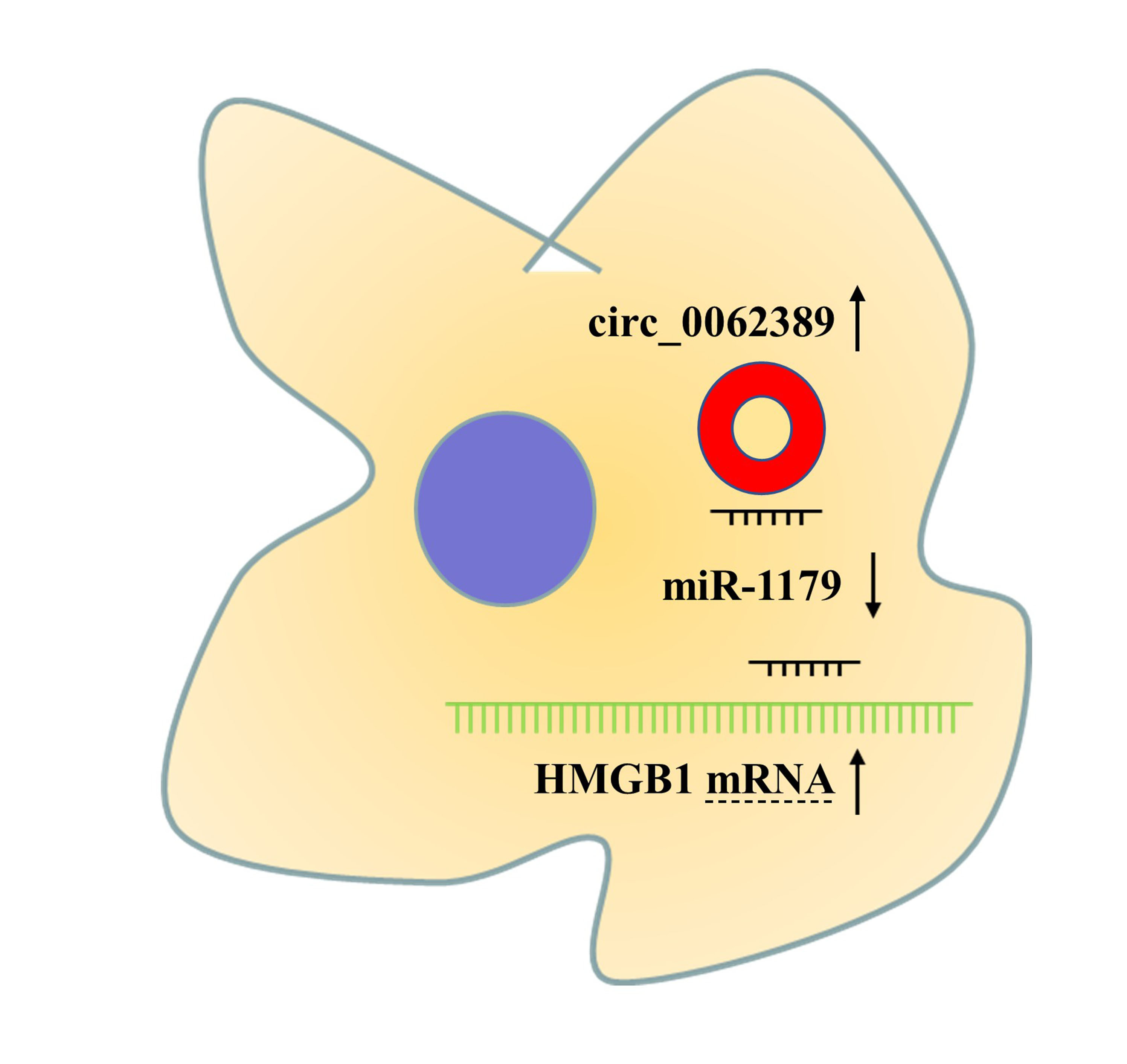

Supplement: Supplemental Material [file KBIE_A_1914470_SM4625.tif]
